# From Stress to Screen: Understanding Cyberloafing through Cognitive and Affective Pathways

**DOI:** 10.3390/bs14030249

**Published:** 2024-03-19

**Authors:** Xinyuan Lu, Yizhou Wang, Xiaoxiao Chen, Quan Lu

**Affiliations:** 1 School of Information Management, Central China Normal University, Wuhan 430079, China; luxy@mail.ccnu.edu.cn (X.L.); cxx915@126.com (X.C.); 2 Hubei Electronic Commerce Research Center, Wuhan 430079, China; 3 School of Economics and Management, Hubei University of Technology, Wuhan 430068, China; luquan@mails.ccnu.edu.cn

**Keywords:** cyberloafing, role stress, conservation of resources theory, cognitive–affective personality systems theory, PLS-SEM

## Abstract

This investigation delves into the pervasive yet insufficiently examined phenomenon of “cyberloafing”, characterized by employees engaging in non-work-related internet activities during office hours. Despite its frequent occurrence in contemporary work environments, the fundamental mechanisms underpinning cyberloafing remain largely uncharted. This study uses the conservation of resources theory and the cognitive–affective personality system framework to demystify the relationship between role stress and cyberloafing. We developed a dual-path model to assess the mediating roles of perceived insider status and emotional exhaustion. Employing SPSS and Smart PLS for data analysis, our research sampled 210 corporate employees. The findings reveal that role stress predicts perceived insider status and emotional exhaustion significantly. Notably, while perceived insider status negatively correlates with cyberloafing, emotional exhaustion shows a positive correlation. These factors mediate the relationship between role stress and cyberloafing, underscoring a multifaceted dynamic. Our results provide new theoretical insights into the mechanisms of employee counterproductive behavior, specifically in the context of cyberloafing, and broaden our understanding of its determinants. This study illuminates theoretical nuances and offers practical implications for managerial strategies and future scholarly inquiries into organizational behavior.

## 1. Introduction

In the digital transformation era, the deep integration of Internet technology within corporate structures has revolutionized work practices and employee interactions with technology. The seamless fusion of digital connectivity into both professional and personal spheres has blurred the lines between work and leisure, leading to frequent employee engagement in personal internet use during work hours, commonly termed ‘cyberloafing’ [1,2]. Since Lim coined the term and defined it as a novel aberrant workplace behavior [3], inception of the term, describing it as a novel aberrant workplace behavior, studies have corroborated that cyberloafing could detrimentally affect employee productivity, performance, and job satisfaction. This is a well-recognized problem that requires a more in-depth study of the underlying mechanisms to provide a scientific basis for interventions by both organizational managers and employees.

Research has traditionally viewed cyberloafing as “counterproductive behavior”, often attributing it to a blend of emotional and cognitive factors [4,5]. Studies focusing on the impact of stressors on cyberloafing have shown that work-related stress often leads to negative emotions, propelling employees towards cyberloafing as a form of emotional relief [6,7,8]. This relationship is further complicated by leadership style, which can significantly influence cyberloafing behaviors by affecting employees’ emotional states [9,10]. Furthermore, cognitive aspects such as organizational commitment and subjective self-control have been identified as critical mediators affecting cyberloafing, demonstrating its complex nature [11,12]. Despite some efforts to explore these emotional and cognitive mechanisms, research on them separately still frequently falls short of capturing the multifaceted nature of cyber-loafing. A holistic understanding of how these dimensions influence cyberloafing behavior is critical and requires a comprehensive approach that includes both affective and cognitive perspectives.

To address this gap, our study introduces an integrated framework that simultaneously examines the cognitive and affective dimensions of stressors influencing cyberloafing. Utilizing the cognitive–affective personality system (CAPS) framework, we explore the pathways through which role stress impacts cyberloafing, considering both cognitive responses like perceived insider status and emotional responses such as emotional exhaustion. This dual-pathway approach provides a more nuanced understanding of cyberloafing and contributes significantly to our knowledge of employee behavior within the modern digital workplace. Our findings aim to inform managerial strategies, offering insights to mitigate the negative impacts of cyberloafing or leverage its potential benefits, fostering sustainable organizational development in the digital era.

## 2. Literature Review and Research Hypothesis

### 2.1. The Relationships between Role Stress and Cyberloafing

In the organizational environment where information technology is deeply embedded in the work network, the issue of role stress in the work environment has gradually received academic attention [13,14]. Social media and virtual interaction platforms have become important ways for employees to communicate, socialize, and share information. People’s social roles have been extended from offline to online, and role stress in traditional workplaces has naturally been extended to online environments [15]. Employees are often faced with an influx of massive amounts of information, while at the same time, the diversity of work tasks and the speed of change continue to accelerate; they need to complete more work in a limited amount of time to meet job requirements and expectations, and role stress has become a heavy burden for employees due to the imbalance arising from the inability to effectively meet the corresponding responsibilities and obligations of the roles [16]. Employees typically have some degree of autonomy over their working hours due to the prevalence of telecommuting and flexible work arrangements. However, this freedom may also lead to employees being more likely to fall into cyberloafing during working hours. They may engage in non-work-related activities such as web browsing and social media browsing during working hours due to the lack of precise time schedules and supervision, which may lead to cyberloafing. The concept of cyberloafing has been more clearly defined by scholars, which refers to employees’ use of the Internet during working hours for non-work-related activities, such as browsing social media, watching videos, and reading news. Although it appears to be a form of escapism and pastime at first glance, cyberloafing can be seen as a way of dealing with role stress. Research indicates that workplace stressors, including role stress, can heighten the likelihood of cyberloafing as a stress relief strategy [17]. Some studies have pointed out that role conflict and ambiguity may lead employees to cyberloafing during working hours to escape from burdensome role demands and unclear expectations. Employees may seek escapism and psychological relief when role stress accumulates, and cyberloafing provides a transient channel for psychological comfort [18]. Other scholars have emphasized the impact of emotional stress on cyberloafing, such as anxiety and frustration, which may prompt employees to seek dynamic relaxation and satisfaction by alleviating emotional discomfort through cyberloafing [19]. In this context, cyberloafing is seen as a self-regulation tool that may help employees temporarily alleviate the discomfort caused by role stress. In addition to the aforementioned perspectives, another viewpoint posits cyberloafing as a manifestation of self-regulation failure during work hours. For example, drawing upon ego depletion theory, Yang et al. [20] propose that employees grappling with feelings of workplace loneliness endeavor to regulate the adverse emotions associated with such loneliness. This emotional regulation effort depletes the employees’ self-regulatory resources, thereby diminishing their self-control capabilities and precipitating a state of ego depletion [21]. Consequently, this condition increases the likelihood of employees engaging in cyberloafing. Synthesizing these research findings, cyberloafing emerges not merely as a strategy to contend with role stress but also as a potential consequence of compromised self-regulatory processes that employees encounter within the complex dynamics of their work environment.

While much of the existing literature on role stress and cyberloafing focuses on role stress as a precursor to increased cyberloafing, an emerging perspective suggests that role stress may also play an inhibitory role in such behaviors. Studies have indicated that subordinates who feel the trust of their superiors often experience increased role stress. Correspondingly, this perception of stress can also make sub-ordinates feel included in their leaders’ “in-group”, recognizing their organizational status [22]. The heightened awareness of role responsibilities and the need to meet performance expectations can provide a counteractive force against counterforce to the allure of cyberloafing. Employees aware of the high-stakes risks of failing to meet job requirements may consciously avoid engaging in non-work-related online activities. This perspective emphasizes role stress, its complex and multifaceted nature, and its impacts on employee behavior.

### 2.2. The CAPS Framework

The cognitive–affective personality system (CAPS) framework theorizes the interplay between individual emotions, cognitions, and behaviors. It posits that situational variables trigger cognitive and affective responses, shaping behavior [23]. This theory systematically explains the relationship between “situation and behavior” based on the “cognitive-affective” dimension of the individual. In this framework, emotion and cognition are intertwined personality traits that influence an individual’s emotional state and impact their behavioral choices and coping strategies. The research hypotheses are categorized into cognitive and affective paths aligning with the CAPS framework. In the cognitive system, multiple social roles in social networks expose employees to frequent communication demands at work, which can help them feel needed by the organization. It can also increase their sense of involvement and belonging within the organization, which in turn can encourage them to stop cyberloafing—something the organization may not consciously allow. In the affective system, however, role stress undoubtedly increases employee workload. As a result, they have to face more complex work requirements, which can consume a lot of their time and energy, resulting in the loss and depletion of adequate resources. Employees then use cyberloafing as a coping strategy to cope with the loss of resources.

Additionally, the conservation of resources theory (COR) will be employed to analyze how cognitive and affective systems, influenced by role stress, contribute to cyberloafing. COR proposes that people always strive to maintain and protect existing resources, acquire and cultivate new resources, and adopt different coping strategies to maintain and accumulate resources [24]. In the cognitive system, employees perceive the insider status as a valuable resource invested in role behaviors to add value to help. This perception inspires employees to develop a stronger emotional connection with the organization, and they may be able to focus more on their work tasks instead of wasting time on irrelevant networking activities. In the affective system, employees will engage in cyberloafing behaviors to alleviate fatigue and reduce resource depletion in the face of emotional deficit, as a lack of resources motivates individuals to protect their valuable resources and reduce the potential loss of resources.

**Figure 1:**
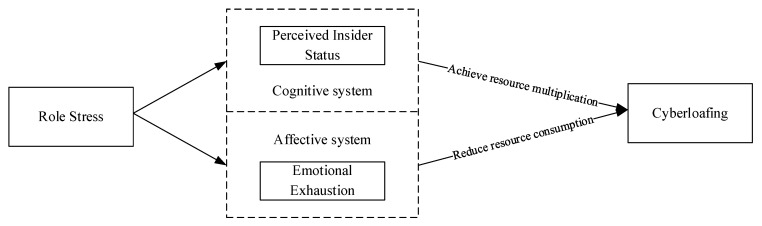
Schematic diagram.

This study integrates the CAPS framework and COR, constructs a schematic diagram of the path of role stress affecting cyberloafing (e.g., Figure 1), and dissects the explanatory roles of the CAPS framework and COR in dealing with the relationship between role stress and cyberloafing, as well as highlighting the theoretical and empirical value of the CAPS framework in this study.

### 2.3. The Mediating Role of Perceived Insider Status

Perceived insider status refers to employees’ perceptions of the scope for development and acceptance they receive as members of an organization [25], which describes employees’ psychological beliefs about their status as members of the organization and is a central reflection of the employee–organization relationship [26]. According to Stamper [27], one way in which organizations differentiate between “insiders” and “outsiders” is through social exchange: organizations offer better treatment to “insiders” and expect them to reciprocate in the form of a reward. Another method is the socialization process, influencing or changing employees’ perceptions of their relationship with the organization. Role stress, as a stressor that often arises during social interactions, is therefore likely to significantly impact perceived insider status.

Role stress describes the dilemmas individuals face during long-term interpersonal interactions within organizations. As a common workplace stressor, role stress arises not only from the leadership of the employee, but any social relationship in the organization may be an antecedent to role stress and perceived insider status, which is a key reflection of the relationship between the organization, can also be probably influenced by role stress. Role stress, especially in social media, poses a dilemma for employees and implies expectations from various social relationships. Over time, employees may feel needed by others in the organization, which signals them that they are an essential part of the organization and, therefore, feel like “insiders”. The feeling of being an “insider” in the organization. It has been pointed out that subordinates’ perception of being trusted by their superiors will bring more role stress to the employees [28]. Accordingly, this perception of pressure also leads to employees feeling included in their “own circle” by their leaders and their status in the organization being recognized.

According to the COR, people always strive to maintain and protect existing resources and acquire and cultivate new ones [24]. Stress arises when individuals realize that their resources are threatened and could be lost, their resources are depleted, or they fail to regain essential resources after making significant investments [29]. As perceived insider status encompasses positive feelings towards the organization, employees will invest in perceived insider status as a resource to achieve a value-added spiral of resources in their role behaviors. On the one hand, employees with high perceived insider status have a strong sense of belonging to the organization and are more willing to build a solid emotional bond with the organization to enhance their relationship with the organization to obtain more resources; in a similar vein, employees with high perceived insider status usually develop a sense of mastery based on the insider identity, and actively and proactively engage in behaviors beneficial to the organization to win the recognition of their leaders and other organizational members. Previous studies have shown that perceived insider status can motivate employees to engage in behaviors that are beneficial to the organization or others, such as constructive behaviors [30], organizational commitment [26], and knowledge sharing [31]. Therefore, this paper argues that the satisfaction of employees’ basic needs by insider status will motivate employees to take the initiative to give back to the organization and reduce cyberloafing. Accordingly, the following hypotheses are proposed:

**H1a.**  *Employees’ role stress is positively related to perceived insider status*.

**H1b.**  *Employees’ perceived insider status is negatively associated with cyberloafing*.

**H1c.**  *Perceived insider status negatively mediates the relationship between role stress and cyberloafing*.

### 2.4. The Mediating Role of Emotional Exhaustion

Emotional exhaustion occurs when an individual’s emotional and associated physiological resources are depleted, usually due to a stress response induced by work stressors [32]. Work demands and pressures consume a large number of individual resources. When the resources consumed by an individual cannot be restored promptly, when an individual’s limited resources are insufficient to complete the work task, or even when the individual’s energy is depleted, it directly affects the employee’s emotions, showing negative states such as fatigue, nervousness, irritability, and frustration. Existing studies have pointed out that role stress, as a typical work stressor, forces employees to continuously give their time and energy, directly triggering emotional exhaustion [33]. Related studies have proved that role stress is the most potent predictor variable of emotional exhaustion [34], which suggests that the continuous accumulation of role stress may induce emotional exhaustion. Role overload reflects that employees perceive that the tasks and requirements they undertake in the same period are beyond their capacity. This overloaded work state will reduce their job satisfaction and increase exhaustion. When employees feel role conflict, they must seek a balance between their “actual role” and their “expected role”. They are overwhelmed, which will overconsume their cognitive and emotional resources and lead to a rapid accumulation of negative emotions; when employees feel role ambiguity, not only do they have to spend extra time and energy searching for information such as work goals but they are also unable to reach specific work requirements, which leads to stress and anxiety and ultimately emotional exhaustion.

Emotional exhaustion, an essential aspect of burnout, correlates with increased cyberloafing, as it serves as an outlet for mitigating workplace stress. According to the COR, a lack of resources prompts individuals to develop a solid motivation to conserve their valuable resources and reduce potential resource losses to preserve the remaining resources and avoid further resource losses [35]. When employees suffer from emotional exhaustion, they actively disconnect from their work and spend their time at work chatting on the Internet and cyberloafing to relieve the fatigue associated with this negative state and mitigate the resulting loss of resources. Cyberloafing can replenish individual resources by acquiring positive emotions and reduce resource consumption by suppressing negative emotions, thereby alleviating emotional exhaustion. Accordingly, the following hypothesis is proposed:

**H2a.**  *Employees’ role stress is positively related to emotional exhaustion*.

**H2b.**  *Emotional exhaustion of employees is positively related to cyberloafing*.

**H2c.**  *Emotional exhaustion positively mediates the relationship between role stress and cyberloafing*.

**Figure 2:**
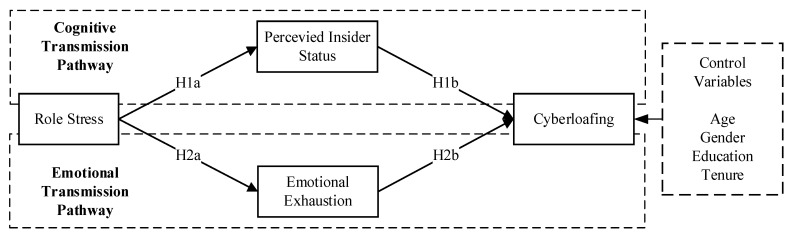
Theoretical model diagram.

This study has developed a research framework, as illustrated in Figure 2. Role stress is posited as the independent variable, while cyberloafing is the dependent variable. Perceived insider status and emotional exhaustion are conceptualized as mediating variables. Through this hypothetical model, the study examines the formation mechanism of employee cyberloafing from the perspective of role stress, grounded in individual cognitive-affective dimensions.

## 3. Method

### 3.1. Participants and Data Collection Procedure

Our survey was distributed online to participants via the “Wenjuanxing” platform, ensuring high anonymity and reliability in collecting sensitive information. Compared to other sampling methods, online platforms like Wenjuanxing have been proven to provide a more diverse sample pool and allow for flexible survey design and high-quality data collection [36]. To ensure that all participants were part of the target demographic for this study, which focuses on the phenomenon of “cyberloafing”—non-productive Internet activities by employees during work hours—several filtering questions were set at the beginning of the survey. For instance, one filtering question was, “Are you currently employed in a corporate setting?” This question helps to confirm that respondents are indeed part of the workforce and potentially exposed to situations where cyberloafing could occur. Another filtering question was, “Do you have regular access to the Internet during your working hours?”. The questionnaire initially outlined the study’s purpose, the involved process, and the participant requirements, clarifying that the gathered data would be used solely for academic research and that participants’ data would be treated as strictly confidential. Attention-check questions were interspersed throughout the questionnaire to ensure response quality. For example, one such question was, “For this item, please select ‘strongly disagree’”. Responses deviating from this instruction were deemed invalid and subsequently excluded from the sample analysis. In total, 225 online questionnaires were collected in this study, with 15 invalid questionnaires (due to incorrect answers to screening questions, too short or too long response time, regular responses, etc.) being excluded to obtain 210 valid questionnaires, resulting in a response rate of 93.3%.

### 3.2. Measurement of Variables

Four variables were identified in this study: role stress, emotional exhaustion, perceived insider status, and cyberloafing. Regarding measuring the variables, the study published by Heggestad et al. [37] provides recommendations for using scales. It suggests that scales in organizational science should be adapted to the context and adopted. Therefore, this study used established domestic and international scales translated and adjusted appropriately to the research context, and the questions were summarized using the two-way translation method to ensure that the intended meaning of the questions was accurately conveyed. The English scale was translated into Chinese using the “translate-back-translate” method. Then, four experts in the field of information systems were invited to review and fill in the pre-test questionnaire. The formal scale was formed after modifying some of the measurement items that were unclear or ambiguous (Appendix A).

#### 3.2.1. Role Stress

Kahn et al. [38] initially introduced the two-factor concept of role stress, including role conflict and role ambiguity, with role overload initially considered a specific and complex form within the domain of role conflict. House et al. later affirmed the reliability and validity of this framework [39]. Subsequent research identified role overload as an independent primary source of role stress, evolving into a three-factor model for measuring role stress. The three-factor role stress scale developed by Peterson et al. [40], building upon Rizzo et al. [41], has been broadly applied and its reliability and stability confirmed through various studies. Recognizing role overload, conflict, and ambiguity as the three core sources of role stress [42], this study utilizes the three-factor role stress scale by Peterson et al., customized to align with the Chinese cultural setting. The scale for this investigation was refined to ensure item clarity and eliminate redundancy.

For instance, role conflict was gauged by items such as “I receive contradictory demands from two or more people”. Role ambiguity was measured by statements like “I clearly understand what different groups expect of my role”. Role overload was evaluated through items such as “Taking on different roles at the same time overburdens me”. Higher scores on these items indicate a higher perceived level of role stress in the corresponding dimension.

#### 3.2.2. Perceived Insider Status

Perceived insider status was measured using the scale developed by Stamper and Masterson [27], with six items, including three reverse-scoring questions. The sample items include “I deeply feel that I am a member of my work organization” or “My work organization often makes me feel abandoned”. The items were rated on a five-point Likert-type scale from one (strongly disagree) to five (strongly agree).

#### 3.2.3. Emotional Exhaustion

Emotional exhaustion was measured using a 4-item scale developed by Maslach and Jackson [43]. Sample items include “My job makes me feel physically and mentally exhausted”. The items were rated on a five-point Likert-type scale from one (strongly disagree) to five (strongly agree).

#### 3.2.4. Cyberloafing

Cyberloafing was measured using Lim and Chen’s 6-item Cyberloafing Behavior Measurement Scale [3]. In line with the specific research context and relevant circumstances in China, some items with similar meanings were merged to streamline the original scales. The original scales, comprising 12 items, were condensed to 6 items. For example, one item assessing cyberloafing behavior was “Online shopping, browsing e-commerce sites, or watching e-commerce live streams for personal reasons”. The response scale ranged from “1”, indicating “rarely”, to “5”, meaning “very frequently”, with higher scores reflecting a higher frequency of cyberloafing behavior.

### 3.3. Data Analysis

According to previous studies, role stress is regarded as a second-order formative type (reflective–formative type) consisting of three reflective first-order variables [44]. Partial least squares structural equation modeling (PLS-SEM) is more applicable when the structural model is more complex and contains formative variables. Secondly, PLS-SEM is advantageous when the data do not necessarily follow a normal distribution, providing robustness against deviations from normality [45]. Finally, PLS-SEM is characterized by its suitability for theoretical development and capability to handle small sample sizes effectively. This study adhered to the evaluation procedures recommended by Hair et al. [46]. Initially, descriptive statistics and correlation analyses were conducted using SPSS 26.0. Subsequently, the measurement model was assessed using Smart PLS 4.0; then, the structural model was evaluated. Finally, the Bootstrapping method within Smart PLS 4.0 was utilized to test for mediation effects.

## 4. Results

### 4.1. Descriptive Statistical Analysis

The demographic breakdown of participants in our study revealed a balanced gender distribution, with males at 51.0% (*n* = 107) and females at 49.0% (*n* = 103). Age categories showed the most significant group was 26–32 years at 41.9% (*n* = 88), with the least being 25 years and below at 16.2% (*n* = 34). Educational levels ranged predominantly from associate degrees at 36.7% (*n* = 77) to Bachelor’s degrees at 42.9% (*n* = 90), while 11.0% (*n* = 23) possessed a Master’s degree or higher. Work tenure varied, with a majority of 55.7% (*n* = 117) within the 2–5 years range and a minority of 2.4% (*n* = 5) exceeding ten years. The survey encompassed employees from various industries, including information technology, finance, consulting, education, construction, and pharmaceuticals. Details of the basic demographic information are shown in Table 1.

### 4.2. Reliability and Validity Test

#### 4.2.1. Reliability Test

The reliability of the constructs in this study was assessed using Cronbach’s alpha, confirming internal solid consistency across all measures. The role conflict, role overload, and role ambiguity scales yielded alphas of 0.850, 0.826, and 0.839, respectively, indicating robust reliability. Perceived insider status and emotional exhaustion demonstrated excellent reliability with alphas of 0.909 and 0.860. Cyberloafing also exhibited good reliability at 0.887. These alpha values surpass the commonly accepted threshold of 0.7, suggesting satisfactory reliability for our scales [47]. The composite reliability (CR) values for all constructs exceeded the threshold of 0.7 [48], further affirming the reliability of the scales used in the study.

#### 4.2.2. Validity Test

Confirmatory factor analysis (CFA) was conducted using SmartPLS 4.0. The results showed that the factor loadings of all question items were higher than 0.7, and all were significant [49] (Table 2); the AVE values of each latent variable ranged from 0.682 to 0.831, which exceeded the criterion of 0.5. In addition, the square roots of the AVE values were all greater than the correlation coefficients of the crossover variables. This indicates that the scale has high convergent and discriminant validity [48] (Table 3). This study also adopted the method proposed by Henseler et al. [45] to assess the discriminant validity based on the HTMT ratio, generally considered less than 0.85, to indicate that the measurement model has good discriminant validity. As shown in Table 4, the HTMT values of all the variables in this study were less than 0.85; therefore, the scale has high discriminant validity.

#### 4.2.3. Common Method Bias Test

Harman’s single-factor method was used to test the effect of standard method bias, and factor analysis was conducted on all the question items, which yielded an unrotated first-factor explanation rate of 15.68%, which is much smaller than 40%, indicating that there is no standard severe method bias problem in this study. In addition to the single-factor test, which existing research suggests may not be sufficiently rigorous, this study further employed the unmeasured latent method constructs (ULMC) approach to assess common method bias [50]. Following the procedure for common method bias testing as suggested by Liang et al. [51], we included in the PLS model a common method factor whose indicators included all the principal constructs’ indicators and calculated each individual’s variances substantively explained by the principal construct and by the method. As indicated in Table 5, the results demonstrated that the average substantively explained variance of the indicators is 0.6806, whereas the average method-based variance is 0.0027. The ratio of substantive variance to method variance is about 252:1. In addition, most method factor loadings are not significant. Consequently, this analysis suggests that common method bias does not pose a significant issue within the scope of the current study.

Since role stress in this study is a second-order formative variable, the variance inflation factor (VIF) was used to test the covariance of all potential variables. The results showed that the VIF values of the variables were less than 3 (Table 2), indicating no significant covariance problems among the variables [45].

### 4.3. Structural Model and Direct Effects

**Figure 3:**
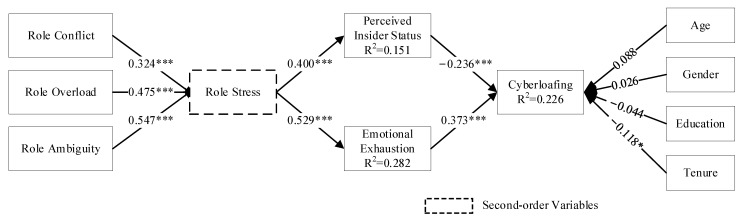
Test results of the structural model. Note: * indicates *p* < 0.05, *** indicates *p* < 0.001.

Since role stress is a second-order variable of reflective-formative type (reflective-formative), structural model analysis was conducted using Smart PLS 4.0 as suggested by Ringle et al. [52], and the results are shown in Figure 3. The standardized residual root mean square (SRMR) of the model in this study was 0.064, which is less than 0.08, indicating that the model’s overall fit is good [44]. The results of the structural model analysis showed that role stress positively affects perceived insider status (β = 0.400, *p* < 0.001), with H1a supported; role stress positively affects emotional exhaustion (β = 0.529, *p* < 0.001), with H2a supported; perceived insider status negatively affects cyberloafing (β = −0.236, *p* < 0.001), with H1b was supported; and emotional exhaustion positively influenced cyberloafing (β = 0.373, *p* < 0.001) and H2b was supported.

### 4.4. Analysis of Multiple Mediating Effects

To test the mediating role of perceived insider status and emotional exhaustion in role stress and employees’ cyberloafing behavior, this study uses the Bootstrap method of Smart PLS 4.0 to verify the hypothesized relationship. It sets the number of repetitive samples to 5000 times and the confidence interval level to 95%, and the results are shown in Table 4. The results show that the confidence interval of perceived insider status is [−0.206, −0.065], which does not contain 0, indicating that the mediating effect of insider status cognition in the process of role stress affecting online loafing behavior is significant. The value of the indirect effect is −0.128, and H1c is verified. Similarly, Table 6 shows that the confidence interval of emotional exhaustion is [0.030, 0.200], not including 0, indicating that emotional exhaustion has a significant mediating effect in the process of role stress affecting online loitering behavior, and the value of the indirect impact is −0.111, H2c is verified.

This paper also refers to the test proposed by Zhao et al. [53] to further determine the type of mediating role of perceived insider status and emotional exhaustion. As shown in Table 6, firstly, the indirect effect of role stress on online loafing behavior (a × b) through perceived insider status is significant (β = −0.128, *p* < 0.001); secondly, the direct effect of role stress on online loafing behavior (c) is significant (β = 0.355, *p* < 0.001); and lastly, the direction of action of the direct and indirect effects are opposite (a × b × c is negative). Thus, perceived insider status plays a competitive mediating role in the effect of role stress on online loafing behavior. Similarly, the indirect effect of role stress on cyberloafing behavior through emotional exhaustion (a × b) was significant (β = 0.111, *p* < 0.01), and the direct and indirect effects acted in the same direction (a × b × c was positive). Thus, emotional exhaustion played a complementary mediating role in the effect of role stress on cyberloafing. Again, H1c and H2c were shown to be validated.

### 4.5. Analysis of Control Variables

The positive control effect of age (β = 0.111, *p* = 0.225) and gender (β = 0.026, *p* = 0.375) on cyberloafing is not significant, and the negative control effect of education (β = −0.044, *p* = 0.375) on cyberloafing is not significant. The negative control effect of work experience (β = −0.118, *p* < 0.05) on cyberloafing behavior was significant, again confirming the study of Lim and Chen [3]. Individual factors such as accumulation of work experience, career achievement, and professional responsibility implied behind the length of service may lead to individuals’ tendency to devote more energy to their careers, which reduces cyberloafing behavior.

## 5. Discussion

In recent years, exploring the multifaceted impacts of role stress has gained traction in academic discourse [8,54,55]. Previous studies have mainly focused on isolating role stress as a significant workplace stressor, influencing organizations and individuals through emotional or cognitive routes [17,18]. Our study departs from this traditional focus and brings to light the simultaneous but opposing influence of both cognitive and emotional pathways. This dual-pathway approach underscores the complex interplay between cognition and emotion in shaping employee behavior in response to role stress. Our findings corroborate the original hypothesis that role stress catalyzes cyberloafing through these two interconnected but distinct mechanisms. This perspective resonates with the insights of Oldham and Cummings [56], who highlighted the complex nature of individual responses to workplace characteristics and emphasized the need to recognize the nuanced and complex responses elicited by workplace dynamics.

Drawing upon our analysis of these mediating effects, we find that perceived insider status is a competitive mediator. Employees who sense a deeper connection with their organization are less inclined to engage in cyberloafing. This cognitive congruence with organizational values and objectives helps employees resist the disruptive lure of role stress, aligning their attention with work-related tasks and reducing the likelihood of cyberloafing as a diversion. Conversely, emotional exhaustion acts as a complementary mediator by amplifying the possibility of cyberloafing among employees grappling with role stress. This affective state—characterized by weariness and burnout—compromises the protective influence of perceived insider status, propelling stressed employees toward cyberloafing as a form of emotional escape. The dichotomy within these findings underscores the complexity of the psychological landscape where employees navigate, suggesting that the emotional impetus for cyberloafing can overshadow the cognitive deterrents associated with feeling valued and integrated within an organization.

Building on the findings of our study, we also consider the broader socio-cultural shifts in the workplace environment. The post-pandemic era has notably altered the landscape of work dynamics, with an increasing emphasis on remote work, digital communication, and flexible schedules. These changes profoundly impact employee behavior, as indicated in our findings and further exemplified by the Great Resignation phenomenon explored in Borrelli et al.’s study [57]. This alignment between cyberloafing as a response to role stress and the broader patterns of employee resignation underlines a common theme of seeking respite from workplace pressures. Traditional approaches focused primarily on monitoring and controlling employee behaviors may no longer be sufficient in the face of these nuanced and complex dynamics. Instead, a more holistic approach that considers employees’ psychological and emotional needs is required. This approach should mitigate cyberloafing and foster an environment where employees feel valued, understood, and supported. Such an environment could potentially reduce the need for employees to seek escape through cyberloafing or resignation, thus enhancing overall productivity and job satisfaction.

### 5.1. Implications for Theory and Research

First, starting from role stress on social networks, this paper enriches the research related to role stress to some extent. It also expands the social motivation sources of online loafing. Tandon et al. [58] pointed out that existing online loafing-related research still lacks the exploration of the motivation of individual online loafing behaviors. This paper combines resource conservation theory and cognitive–affective personality system theory, constructs a dual-path influence model, explores the influence of role stress on online loitering behavior, and analyzes the role of two different paths, namely, the positive role of role stress at the level of individual cognition and the negative role of role stress at the level of personal emotion, to provide ideas for the study of the generation of online loitering behavior. This provides ideas for the study of the mechanism of cyberloafing. In addition, previous studies have focused on the adverse effects of role stress, with little attention paid to the potential positive effects of role stress, and this integration can help to deeply understand the psychological mechanisms of individuals’ cyberloafing under role stress.

Second, exploring the mediating role of perceived insider status and emotional exhaustion between role stress and cyberloafing provides empirical support for understanding how role stress influences cyberloafing. This study helps to reveal the mechanisms by which role stress influences cyberloafing and enriches the application of resource conservation theory and cognitive–affective personality systems theory to the work environment.

### 5.2. Implications for Practice

This study provides valuable insights into corporate management practices, which can help to better understand and cope with employees’ possible cyberloafing in the work environment, especially in the face of role stress.

(1) Role stress management and strengthening insider status: Employees should be aware of the potential positive attributes of role stress, recognize that role stress is often accompanied by closer relationships, greater power, or higher status, and then actively channel the positive effects of these pressures by adjusting their mindset to mitigate their negative impact. It also strengthens the sense of identity within the organization and enhances performance and self-involvement in the role. Business managers must intervene with employees who feel higher levels of role stress, e.g., by providing more explicit role expectations, reasonable work assignments, and better support systems, which can reduce the role stress generated by employees during their work. Reducing role stress can help reduce employees’ likelihood of adopting cyberloafing, improving work efficiency and performance.
(2) Provide emotional support and stress management training: Business managers can focus on the pressures and expectations of employees in different roles and take steps to reduce role stress. For example, helping employees better adapt to the demands of their roles by providing support, training, and resources can help reduce the risk of emotional exhaustion. On the other hand, given the positive association between emotional exhaustion and cyberloafing, organizations may consider providing employees with emotional support and training on coping with stress to help them better cope with role stress and effectively manage emotions and feelings of stress, to help employees learn to handle and cope with their emotions effectively to reduce undesirable cyberloafing.
(3) Promote positive cyberloafing behaviors: Enterprises can encourage employees to use network resources for knowledge acquisition, information exchange, and work collaboration under appropriate circumstances to meet work demands better. Providing appropriate network resources and platforms and promoting positive network use behaviors will help employees better cope with role stress and reduce unnecessary network loitering behaviors.

### 5.3. Limitations and Future Research

Although this study has obtained some valuable findings on the relationship between role stress and cyberloafing, there are still some shortcomings, and future research can explore and deepen the following aspects:

(1) This study mainly focuses on the individual level. However, inducing and influencing cyberloafing may also be affected by organizational factors, such as leadership style, organizational climate, and interpersonal relationships. Therefore, future research can further explore how organizational-level factors influence online loitering behavior.
(2) Regarding generalizability, our findings hold significant implications for various industries and organizational settings, given the ubiquitous nature of role stress and cyberloafing in the digital era. The sampling validity of our study, which spans multiple sectors and job roles, contributes to the robustness and ecological validity of our conclusions. However, the potential biases, such as self-reporting in our measurement protocols and the inherent limitations of the study design, must be acknowledged as they may influence the internal and statistical validity of our findings. Furthermore, while our sample size was adequate for the analytical methods employed, future research could benefit from more extensive and diverse populations to enhance the external validity of these findings.

## 6. Conclusions

This study delved into the complex dynamics of role stress and its impact on employee cyberloafing behavior. By applying the CAPS framework and COR, our study initially constructed the link between role stress and cyberloafing, revealing that role stress influences cyberloafing behavior in a dual-edged manner through two competitive mediators: perceived insider status and emotional exhaustion. These findings enrich our understanding of how employees navigate complex work environments, balancing cognitive assessments and emotional responses. Thus, this study goes beyond the traditional knowledge of cyberloafing, offering a holistic view of its relationship with role stress.

## Figures and Tables

**Table 1:** Demographic.

Variable	Category	Frequency	Proportion/%
Gender	Male	107	51.0
	Female	103	49.0
Age	25 and below	34	16.2
	26–32 years	88	41.9
	33–39 years	56	26.7
	40 and above	32	15.2
Education	High school and below	20	9.5
	Associate degree	77	36.7
	Bachelor’s degree	90	42.9
	Master’s degree and above	23	11.0
Tenure	Less than two years	60	28.6
	2–5 years	117	55.7
	5–10 years	28	13.3
	Over ten years	5	2.4

**Table 2:** Reliability and validity test results.

Variable	Dimension	Item	Standard Factor Loadings	VIF	Cronbach’s α	CR	AVE
Role Stress (RS)	Role Conflict	RC1	0.806	2.329	0.841	0.850	0.759
		RC2	0.800	0.841			
		RC3	0.766	2.004			
	Role Overload	RO1	0.756	2.089	0.824	0.826	0.740
		RO2	0.768	1.710			
		RO3	0.756	1.895			
	Role Ambiguity	RA1	0.791	2.031	0.832	0.839	0.748
		RA2	0.774	1.736			
		RA3	0.837	2.100			
Perceived Insider Status (IS)		IS1	0.728	2.101	0.899	0.909	0.663
		IS2	0.764	1.967			
		IS3	0.794	2.304			
		IS4	0.846	2.297			
		IS5	0.789	2.136			
		IS6	0.711	2.032			
Emotional Exhaustion (EE)		EE1	0.724	1.819	0.860	0.862	0.640
		EE2	0.724	1.793			
		EE3	0.730	1.779			
		EE4	0.730	1.888			
		EE5	0.768	2.037			
Cyberloafing (CL)		CL1	0.757	1.947	0.881	0.887	0.627
		CL2	0.727	1.845			
		CL3	0.727	1.829			
		CL4	0.747	2.049			
		CL5	0.732	1.979			
		CL6	0.742	2.122			

**Table 3:** Discriminant validity test results (Fornell–Larcker criteria).

Variable	Role Conflict	Role Overload	Role Ambiguity	Perceived Insider Status	Emotional Exhaustion	Cyberloafing
Role Conflict	(0.871)					
Role Overload	0.352 **	(0.860)				
Role Ambiguity	0.329 **	0.257 **	(0.865)			
Perceived Insider Status	0.156 *	0.361 **	0.293 **	(0.814)		
Emotional Exhaustion	0.429 **	0.419 **	0.318 **	0.289 **	(0.800)	
Cyberloafing	0.236 **	0.273 **	0.230 **	−0.128 **	0.297 **	(0.792)

Note: * indicates *p* < 0.05, ** indicates *p* < 0.01. The numbers in parentheses on the diagonal represent the square root of AVE.

**Table 4:** Discriminant validity test results (heterotrait–monotrait ratio).

Variable	Role Conflict	Role Overload	Role Ambiguity	Perceived Insider Status	Emotional Exhaustion	Cyberloafing
Role Conflict						
Role Overload	0.423					
Role Ambiguity	0.393	0.312				
Perceived Insider Status	0.180	0.422	0.339			
Emotional Exhaustion	0.506	0.496	0.378	0.331		
Cyberloafing	0.274	0.319	0.268	0.156	0.342	

**Table 5:** Common method bias analysis.

Construct	Indicator	Substantive Factor Loading (*R*1)	*R*1^2^	Method Factor Loading (*R*2)	*R*2^2^
Role Stress	RA1	0.8740 **	0.7639	−0.0090	0.0001
	RA2	0.8380 **	0.7022	0.0150	0.0002
	RA3	0.8830 **	0.7797	−0.0460	0.0021
	RC1	0.8980 **	0.8064	0.1050	0.0110
	RC2	0.8480 **	0.7191	0.1390 *	0.0193
	RC3	0.8670 **	0.7517	0.0510	0.0026
	RO1	0.8850 **	0.7832	−0.0240	0.0006
	RO2	0.8340 **	0.6956	−0.0790	0.0062
	RO3	0.8600 **	0.7396	−0.0250	0.0006
Emotional Exhaustion	EE1	0.7940 **	0.6304	0.0370	0.0014
	EE2	0.7880 **	0.6209	−0.0590	0.0035
	EE3	0.7850 **	0.6162	0.0140	0.0002
	EE4	0.8060 **	0.6496	−0.0220	0.0005
	EE5	0.8280 **	0.6856	−0.0980	0.0096
Perceived Insider Status	IS1	0.8140 **	0.6626	−0.0400	0.0016
	IS2	0.7950 **	0.6320	0.0080	0.0001
	IS3	0.8350 **	0.6972	−0.0080	0.0001
	IS4	0.8320 **	0.6922	−0.0020	0.0000
	IS5	0.8140 **	0.6626	−0.0440	0.0019
	IS6	0.8000 **	0.6400	−0.0170	0.0003
Cyberloafing	CL1	0.7860 **	0.6178	−0.0310	0.0010
	CL2	0.7700 **	0.5929	0.0590	0.0035
	CL3	0.7750 **	0.6006	0.0550	0.0030
	CL4	0.8040 **	0.6464	−0.0210	0.0004
	CL5	0.7990 **	0.6384	−0.0030	0.0000
	CL6	0.8170 **	0.6675	0.0370	0.0014
Average		0.8242	0.6806	−0.0003	0.0027

Notes: * *p* < 0.05; ** *p* < 0.01.

**Table 6:** Bootstrap test results of mediating effect.

Effect	Path	Path Coefficient	T Value	95% Confidence Interval
Direct effects	RS → CL	0.355 ***	4.401	[0.201, 0.517]
Indirect effects	RS → IS → CL	−0.128 ***	3.564	[−0.206, −0.065]
	RS → EE → CL	0.111 **	2.58	[0.030, 0.200]

Note: ** indicates *p* < 0.01, *** indicates *p* < 0.001.

## Data Availability

The data are available upon request from the corresponding author.

## Appendix A

### Survey Questionnaire

Please choose the option that best matches your true feelings at work and mark it with a “√”. 1–5 represents “Completely Disagree” to “Completely Agree”, where a higher number indicates a greater degree of agreement.

**Table A1:** Measurement of Role Stress.

Number	Item	Completely Disagree	Somewhat Disagree	Uncertain	Somewhat Agree	Completely Agree
1	I receive contradictory demands from two or more people.	1	2	3	4	5
2	I often face situations with conflicting demands.	1	2	3	4	5
3	Sometimes I have to violate some role behavior norms to do what I want.	1	2	3	4	5
4	I clearly understand what different groups expect of my role.	1	2	3	4	5
5	The roles I undertake have clear, planned goals and purposes.	1	2	3	4	5
6	When taking on different roles, I know what my responsibilities are.	1	2	3	4	5
7	Taking on different roles at the same time overburdens me.	1	2	3	4	5
8	I have so many roles that I can’t cope easily.	1	2	3	4	5
9	The expectations of different roles leave me overwhelmed, affecting my work quality.	1	2	3	4	5

**Table A2:** Measurement of Perceived Insider Status.

Number	Item	Completely Disagree	Somewhat Disagree	Uncertain	Somewhat Agree	Completely Agree
1	I deeply feel that I am a member of my work organization.	1	2	3	4	5
2	My work organization makes me believe that I am part of it.	1	2	3	4	5
3	I feel that I am an insider in my work organization.	1	2	3	4	5
4	I feel like an “outsider” in the organization.	1	2	3	4	5
5	I don’t feel like I belong to this organization.	1	2	3	4	5
6	My work organization often makes me feel abandoned.	1	2	3	4	5

**Table A3:** Measurement of Emotional Exhaustion.

Number	Item	Completely Disagree	Somewhat Disagree	Uncertain	Somewhat Agree	Completely Agree
1	My job makes me feel physically and mentally exhausted.	1	2	3	4	5
2	After a whole day of work, I feel completely drained.	1	2	3	4	5
3	Waking up in the morning and thinking about facing a day of work makes me feel exhausted.	1	2	3	4	5
4	Working all day is indeed very stressful for me.	1	2	3	4	5
5	Work makes me feel on the verge of a breakdown.	1	2	3	4	5

**Table A4:** Measurement of Cyberloafing.

Number	Item	Completely Disagree	Somewhat Disagree	Uncertain	Somewhat Agree	Completely Agree
1	Receiving, checking, or sending personal emails.	1	2	3	4	5
2	Browsing non-work-related websites (such as news, sports, entertainment).	1	2	3	4	5
3	Using instant messaging software (such as WeChat, QQ) for non-work-related matters.	1	2	3	4	5
4	Online shopping, browsing e-commerce sites, or watching e-commerce live streams for personal reasons.	1	2	3	4	5
5	Online job searching, browsing recruitment websites, or looking for job-hopping opportunities, etc.	1	2	3	4	5
6	Playing online games (such as mini-games, browser games).	1	2	3	4	5
